# Supplementary material for: An Ultrasensitive and Selective Determination of Cadmium Ions at ppt Level Using an Enzymic Membrane with Colorimetric and Electrochemical Detection
Source: Biosensors (Basel). 2022 May 7;12(5):310. doi: 10.3390/bios12050310 (PMC9138971; doi:10.3390/bios12050310)
Supplement: Supplementary file 1 [file biosensors-12-00310-s001.zip › biosensors-1694380-supplementary.pdf]

Supplementary material

An Ultrasensitive and Selective Determination of Cadmium Ions at Ppt Level Using an Enzymic Membrane with Colorimetric and Electrochemical Detection

Raouia Attaallah and Aziz Amine *

Laboratory of Process Engineering and Environment, Faculty of Sciences and Techniques, Hassan II University of Casablanca, Mohammedia 21100, Morocco; raouia.attaallah-etu@etu.univh2c.ma

* Correspondence: azizamine@yahoo.fr or a.amine@univh2m.ac.ma

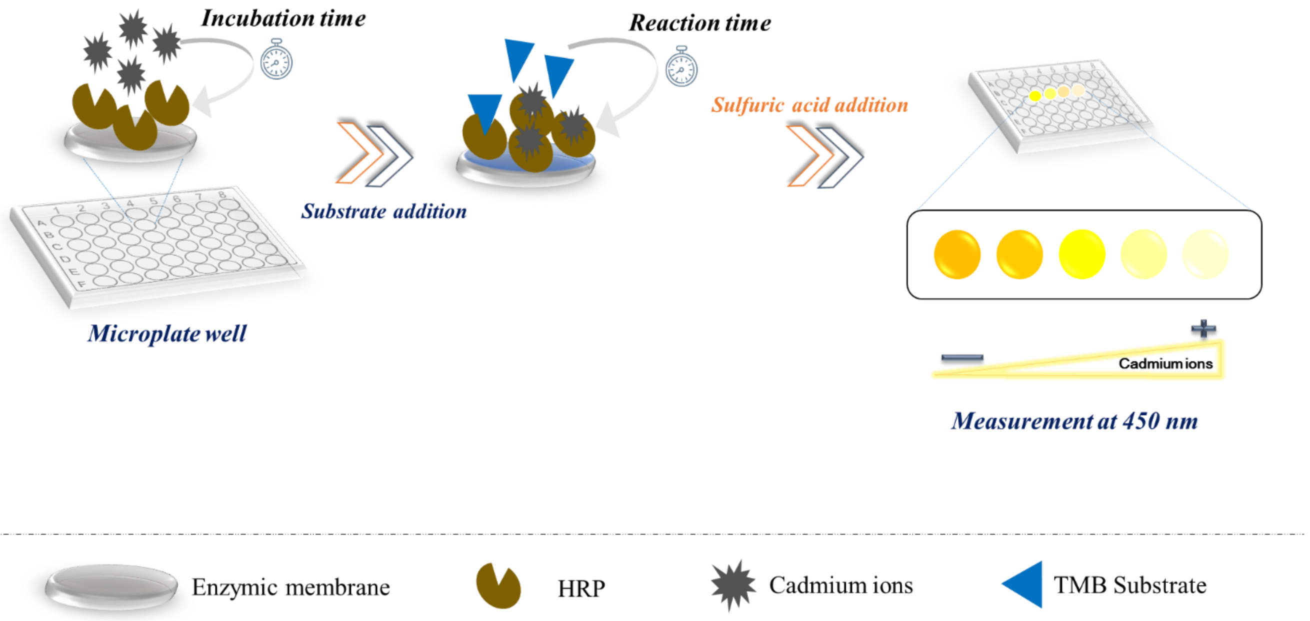

Scheme S1. Schematic illustration of the developed spectrophotometry assay based on enzymic membrane for cadmium ions detection.

(A)

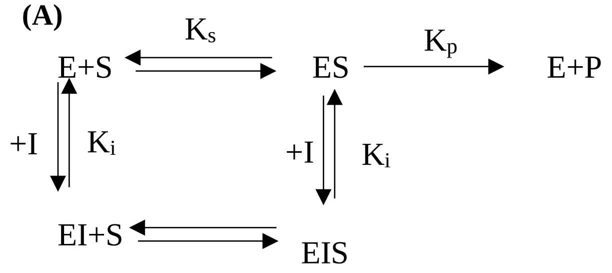

(B)

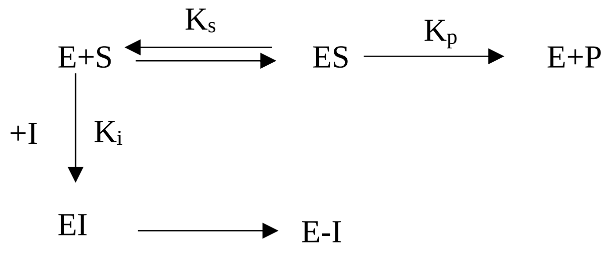

Scheme S2. Scheme of enzyme inhibition in the case of (A) reversible inhibition and (B) irreversible inhibition

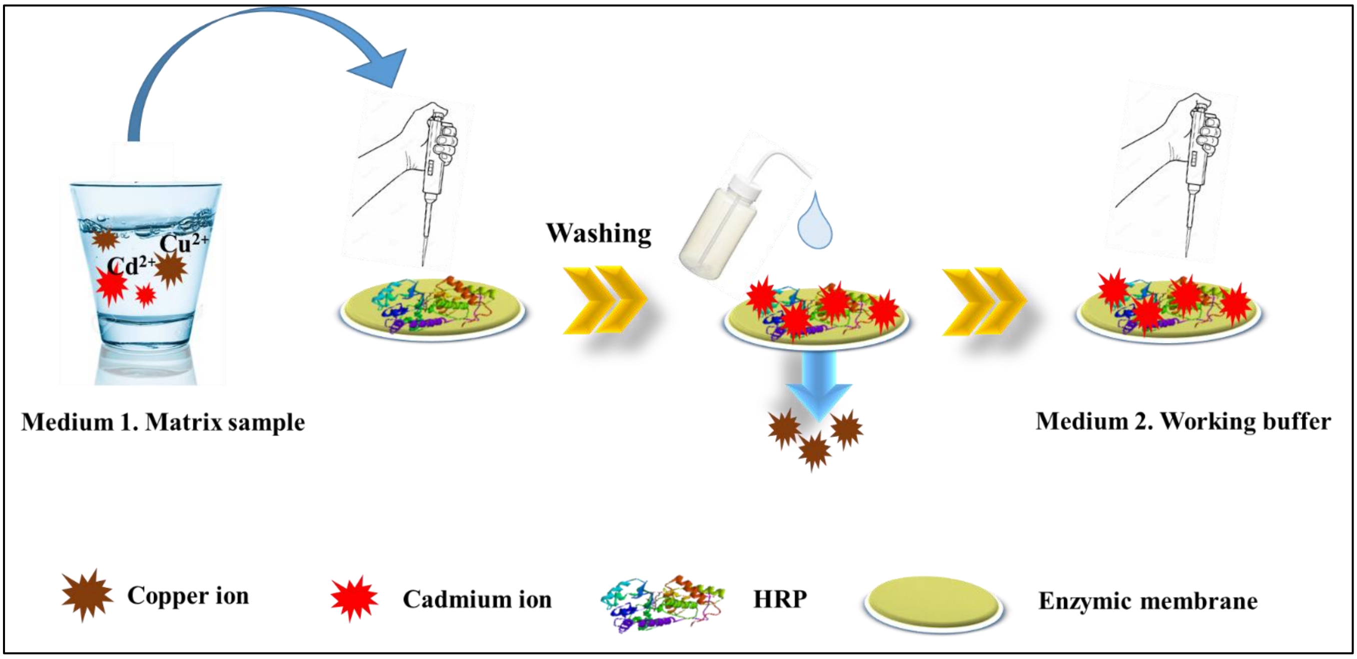

Scheme S3. Schematic illustration of medium exchange procedure.

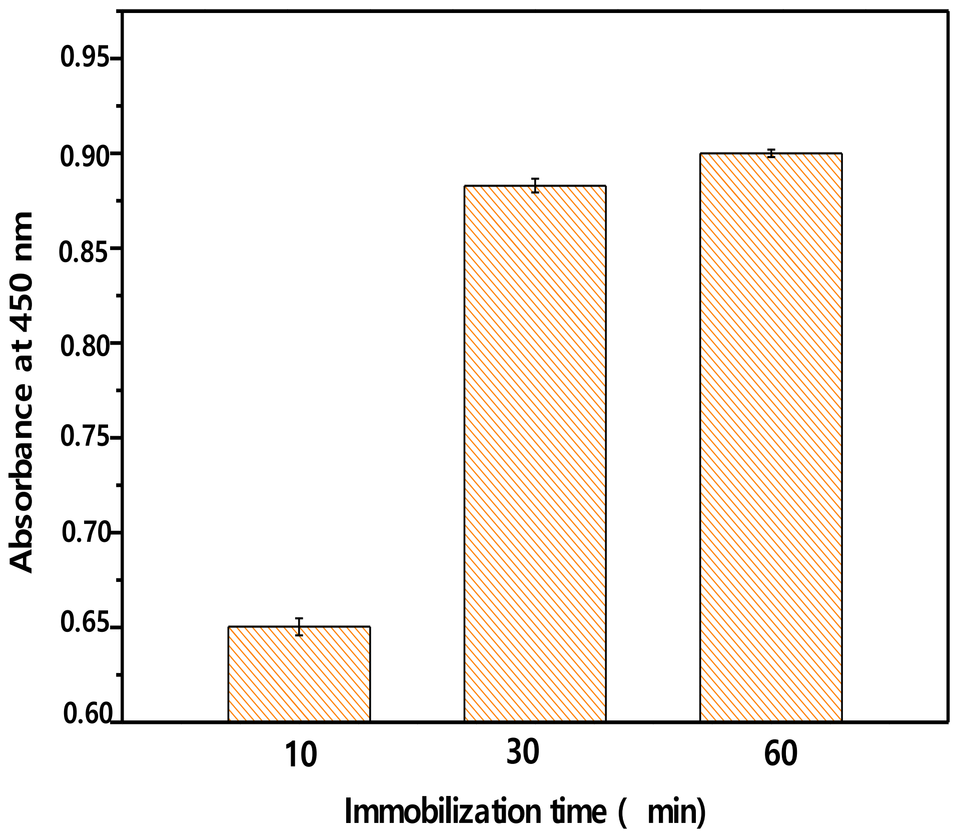

Figure S1. effect of immobilization time on the enzyme loading on the membrane. Data are mean ± SD, n = 5, RSD=2 %

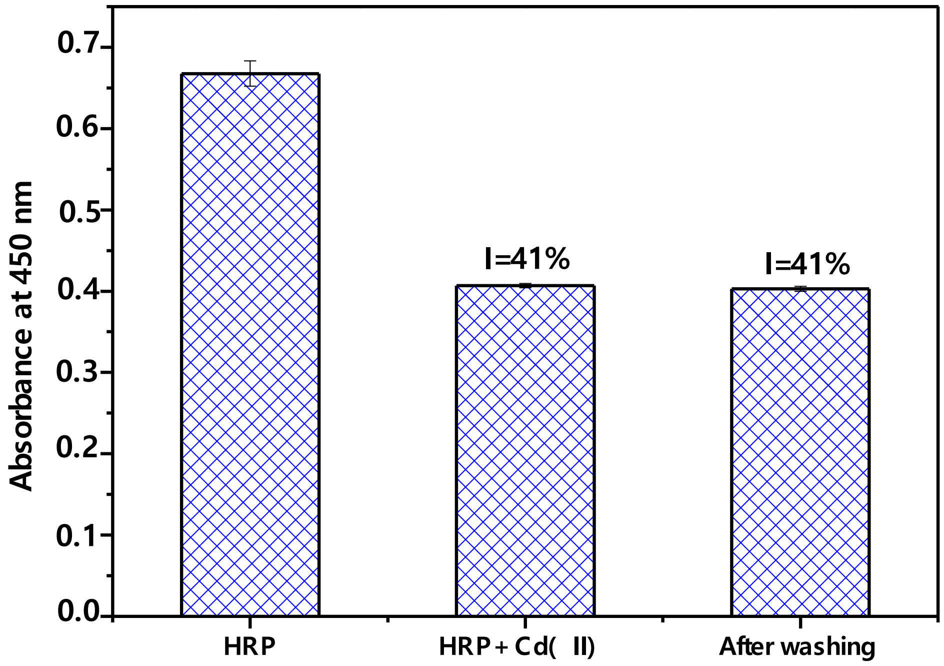

Figure S2. The absorbance response of the enzymic membrane when incubated with 10 ppb Cd^{2+}. Data are mean ± SD, n = 5.

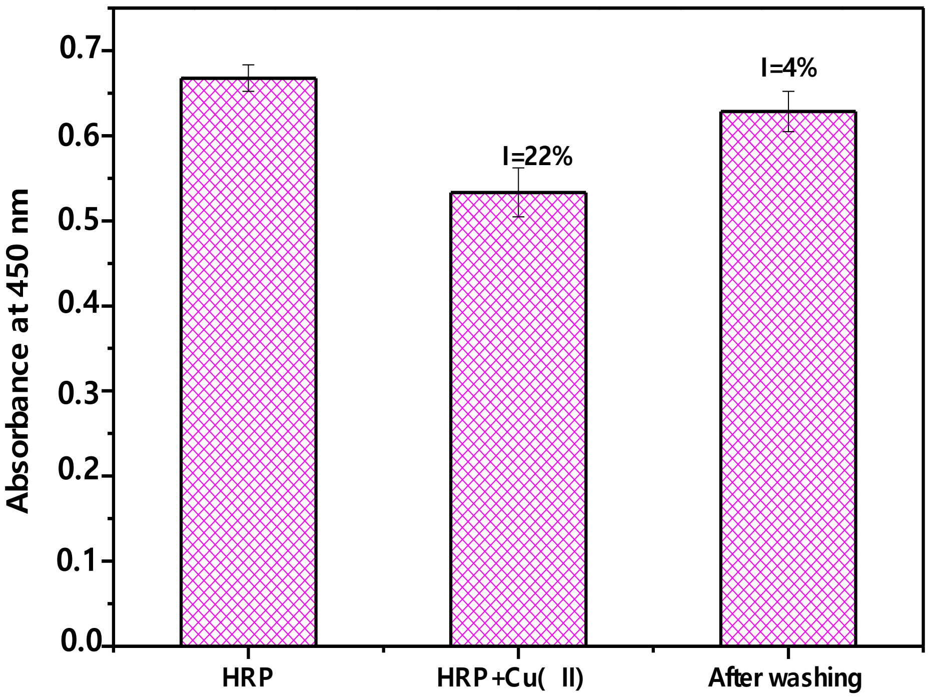

Figure S3. The absorbance response of the enzymic membrane when incubated with 50 ppm Cu^{2+}. Data are mean ± SD, n = 5.

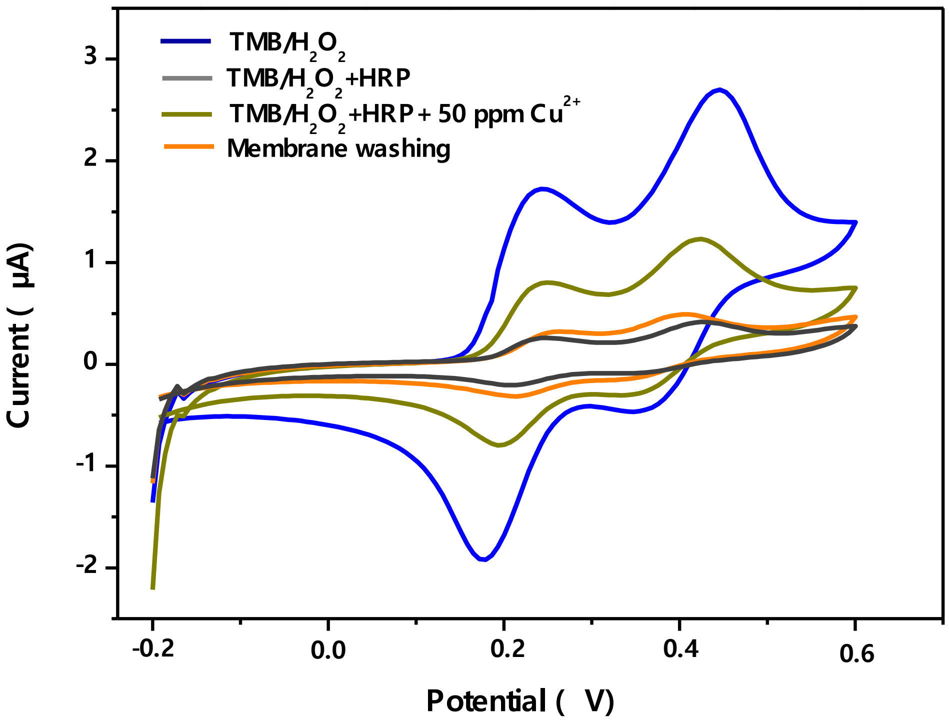

Figure S4. Cyclic voltammograms for the response of the biosensor when incubated before and after 50 ppm Cu^{2+}.
